# The Synthesis of α-MoO_3_ by Ethylene Glycol

**DOI:** 10.3390/ma6104609

**Published:** 2013-10-17

**Authors:** Tzu Hsuan Chiang, Hung Che Yeh

**Affiliations:** Department of Energy Engineering, National United University, 1, Lienda, Miaoli 36003, Taiwan; E-Mail: kevin25831@yahoo.com.tw

**Keywords:** ethylene glycol, molybdenum trioxide, crystallization, sintering

## Abstract

This study investigated the use of ethylene glycol to form α-MoO_3_ (molybdenum trioxide) from ammonium molybdate tetrahydrate at various sintering temperatures for 1 h. During the sintering process, the morphologies of the constituents were observed using scanning electron microscopy (SEM), and Fourier transform infrared (FTIR) spectroscopy was used to explain the reaction process. In this work, the results obtained using X-ray photoelectron spectroscopy (XRD) demonstrated that, when the molybdenum trioxide powder was treated thermally at 300 °C, the material exhibited crystallinity. The peaks were indexed to correspond with the (110), (040), (021), (111), and (060) crystallographic planes, and the lattice parameters of a, b, and c were about 3.961, 13.876, and 3.969 Å. Using these observations, we confirmed that orthorhombic α-MoO_3_ was formed for sintering temperatures from 300 to 700 °C. Pattern images were obtained by the selected area electron diffraction pattern (SAED) technique, and the d distance of the high resolution transmission electron microscopy (HRTEM) images were almost 0.39 and 0.36 nm, and the Mo 3d_5/2_, Mo 3d_3/2_, and O 1s of X-ray photoelectron spectroscopy (XPS) were located at 233.76, 237.03, and 532.19 eV, which also demonstrated that α-MoO_3_ powder had been synthesized.

## 1. Introduction

Molybdenum trioxide has received considerable attention over the last few years because of its many applications in various fields. The various forms of molybdenum trioxide have several important properties that have contributed to this attention, such as good photocatalytic ability [1], battery device assembly and Li storage performance [2]. Therefore, MoO_3_ is used extensively in industry in the catalysts [3], in the field effect transistors [4], in the gas sensors [5] and in the electrodes of batteries [6].

Molybdenum trioxide and its derivatives have been synthesized by a variety of physical and chemical manufacturing methods. In addition, Balendhran *et al.* have classified these methods into three major categories: vapor, liquid and solid phase deposition techniques [7]. These products have been synthesized by various chemical methods, including electrodeposited [8], thermal evaporation [9], poly(ethylene oxide)-containing polymers with peroxomolybdate solutions [10], chemical vapor deposition (CVD) [11], chemical vapor transport of volatile MoO_3_(OH)_2_ [12], the sol-gel process [13], evaporation of HNO_3_ [14], HCl chemical precipitation [15], directly oxidation of a spiral coil of molybdenum [16], NaCl-assisted hydrothermal treatment [17], HNO_3_ and H_2_O_2_ [18], polyethylene glycol [19], the hydrothermal-sol gel method [20], the acid-base titration method [21], electrochemical processes [22], cation-exchange resins [23], micelles of a PEO–PBO–PEO [PEO, poly(ethylene oxide) triblock copolymer [24], solution combustion method [25], ethylene diamine tetra-acetic acid (EDTA), ammonium molybdate and alkali metal molybdate with ethylene glycol as catalysts for the reaction of propylene with an organic hydroperoxide [26].

In addition to the above research, ethylene glycol has not been used to react with ammonium molybdate tetrahydrate, which is a new chemical method for the synthesis of α-MoO_3_. The purpose of this work was to establish and optimize the conditions for the production of α-MoO_3_ powder by ethylene glycol. In our work, we used ethylene glycol mixed with ammonium molybdate tetrahydrate, and we found that α-MoO_3_ powder could be formed from this relatively simple combination of reactants. The results that we achieved were dependent on the times and the temperatures used in the sintering process. 

## 2. Results and Discussion

### 2.1. Effect of the Content of the Ethylene Glycol 

Two hundred fifty milliliters of a 0.1 M ammonium molybdate tetrahydrate solution were mixed with various contents of the ethylene glycol, *i.e.*, 25, 50, 100 and 125 mL and heated to a temperature of 120 °C for 40 min. The results display different blue color for various contents of the ethylene glycol. Table 1 shows the reacted ratio (*R*) of Mo that was determined from the residual Mo content in the each sample after the blue liquid was removed. The *R* of Mo was calculated as shown [27]:

*R*(%) = ([Mo]_0_ − [Mo]_f_)/[Mo]_0_ × 100 (1)

where [Mo]_0_ is the initial Mo content in the solution, and [Mo]_f_ is the Mo content after the removal of the blue product produced by the reaction. The largest *R* of Mo ions was 64.2% when 50 mL of the ethylene glycol was heated at 120 °C for 40 min, as shown in Table 1. At 100 mL, the *R* was decreased to 42%, and, at 125 mL, the *R* was decreased to 17.7%. This was because the excess ethylene glycol prevented the reaction of the molybdenum ions and required in order that the molybdic acid reagent will be utilized to a satisfactory extent at 120 °C. Therefore, the reaction temperature should be increase that can helpful to produce a precipitate.

**Table 1:** Reacted ratio of the Mo and residual Mo concentration for different amounts of the ethylene glycol.

0.1 M of ammonium molybdate tetrahydrate solution (mL)	Ethylene glycol (mL)	Mo (ppm)	*R* (%)
250	–	85,960	–
	25	41,920	51.2
	50	30,764	64.2
	100	49,840	42
	125	70,630	17.8

### 2.2. Effect of Sintering Temperatures

**Figure 1:**
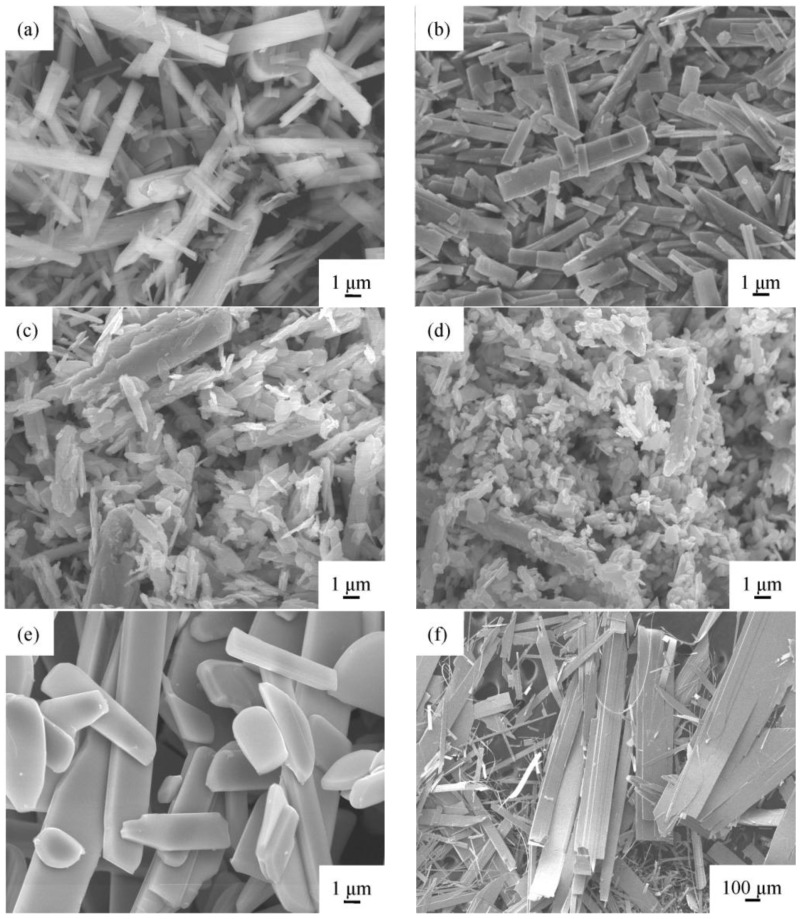
SEM images of molybdenum trioxides before sintering and after sintering at various temperatures: (**a**) without sintering; (**b**) 200 °C; (**c**) 300 °C; (**d**) 500 °C; (**e**) 600 °C; (**f**) 700 °C.

Fifty milliliters of ethylene glycol were mixed with 250 mL of a 0.1 M ammonium molybdate tetrahydrate solution, which was heated for 40 min at 120 °C, producing molybdenum trioxide powder. The particle sizes and morphologies of the MoO_3_ powder that resulted from sintering at 300 °C, 500 °C, 600 °C, and 700 °C for 1 h are shown in the SEM images in Figure 1. Since the melting point of MoO_3_ is 795 °C, we did not exceed the sintering temperature of 700 °C. Compared to the particle size of the non-sintered MoO_3_ powder, the particle sizes decrease with sintering temperatures up to 500 °C. The shape of the MoO_3_ particles was rectangular before sintering and after sintering at a temperature of 200 °C. When the sintering temperature was increased to 300 °C, the shape of the particles became irregular. The particle size ranged from about 200 to 300 nm, and the particles became rice-shaped at a sintering temperature of 500 °C. At 600 °C, the particles assumed a block, sheet-like structure with a thickness of about 500 nm. At 700 °C, the particles formed piled sheets with nanometer-scale thicknesses. Clearly, the sizes and shapes of the particles can be regulated by controlling the sintering temperature.

**Figure 2:**
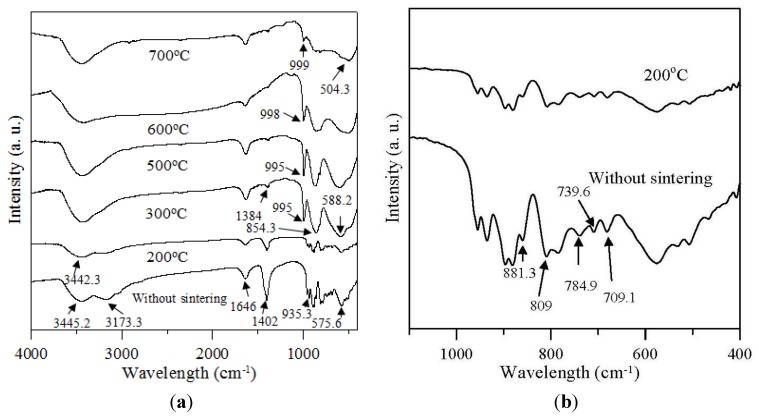
FTIR spectra: (**a**) MoO_3_ powders before sintering and after sintering for 1 h at various temperatures up to 700 °C measured in the 400–4000 cm^−1^ range; (**b**) MoO_3_ powders before sintering and after sintering for 1 h at 200 °C measured in 400–1100 cm^−1^ range.

FTIR spectroscopy was performed to investigate the chemical bonding states between the molybdenum and oxygen atoms in MoO_3_ particles that had different structures at different sintering temperatures. Figure 2a shows the FTIR spectra (measured in the 400–4000 cm*^−^*^1^ range) for the particles that had not been sintered and those that had been centered for 1 h at the various temperatures up to 700 °C. Figure 2b, shows the FTIR spectra (measured in the 400–1100 cm*^−^*^1^ range) for MoO_3_ particles before they were sintered and after they were sintered for 1 h at 200 °C. These spectra clearly indicate the change in the peak that occurred between these two conditions. In Figure 2b, the peak that occurred at 709.7 cm^−1^ was (*v*Mo–O) and (*δ*Mo–O) vibrations, and the peaks at 784.9 and 809 cm^−1^ can be assigned to (*v*Mo–O) vibrations. The peaks between 750 and 1000 cm^−1^ gave the most conclusive information on the change of the Mo–O coordination [28], and the peak at 881.3 cm^−1^ in the range of 875–885 cm^−1^ was attributed to the Mo–O–Mo vibrations of Mo^6+^ [13,28] for the untreated powder and for the powder after sintering for 1 h at 200 °C. When powders were sintered for 1 h at 300 °C and above, the peaks at 709.7, 739.6, 784.9, and 809 cm^−1^ were no longer present, and the peak at 881.3 cm^−1^ was shifted to 854.3 cm^−1^ when the sintering temperature increased from 200 to 300 °C, as shown in Figure 2a. The presence of a single absorption peak at 854.3 cm^−1^ implied that the lengths of the Mo–O bonds on both sides of the O in Mo–O–Mo were symmetrical as the result of the two MoO_6_ octahedra having a corner-shared oxygen in common. However, the intensity of the peak at 854.3 cm^−1^ decreased as the sintering temperature was increased from 500 to 700 °C, indicating that the stretch peak of the terminal oxygen was resolved in this range sintering temperatures. The peak at 575.6 cm^−1^ also was shifted to 588.2 cm^−1^ as the sintering temperature was increased from 300 to 500 °C, and the peak at 588.2 cm^−1^ was assigned to the stretching mode of the triply-coordinated oxygen (Mo_3_–O), which results from the edge-shared oxygen in common with three MoO_6_ octahedra [29]. In addition, when sintering temperatures were increased to 600 and 700 °C, the peak at 588.2 cm^−1^ was no longer present, and the peak that occurred at 504.3 cm^−1^ was attributed to the O–Mo–O deformation mode, a result that was the same as that reported by Pereira *et al.* [30] In Figure 2a, the bands located at 935.3 cm^−1^ (*v*Mo–O–Mo) for the powder that had not been thermally treated were shifted to the range of 995.1–999 cm^−1^ (*v*Mo = O) for the powder that had been sintered at temperatures up to 300 °C, and the presence of terminal double bonds was a basic characteristic of the layered, orthorhombic MoO_3_ phase [31]. But the intensity of the peaks decreased as sintering temperature was increased from 300 to 700 °C because the stretch peak of the terminal oxygen was resolved, causing the structural change. 

The two peaks that were detected at 1402 (*δ*NH_4_^+^) and 3173.3 cm*^−^*^1^ (*v*NH_4_^+^) represented the residual amine group (NH_4_^+^) of ammonium molybdate in the powder without thermal treatment, and the peak at 3173.3 cm^−1^ no longer existed and, simultaneously, the intensity of the peak at 1402 cm^−1^ decreased when the powder was sintered for 1 h at 200 °C. This was the result of the NH_4_^+^ groups forming NH_3_ gas that was released from the powder at the higher temperature [32]. In addition, the vibrations of the two peaks were detected at 1384.6 cm^−1^, and they were associated with the vibration mode of the *δ*Mo–OH bending vibrations (1000–1491 cm^−1^) [33] and 1644 cm*^−^*^1^ (sintering temperature up to 300 °C) and 1646 cm^−1^ (powder without thermal treatment), associated with the *δ*H_2_O bending vibrations, respectively. The results were the same as those reported by Dhanasankar *et al.* [34]. The (*v* OH) absorption band is very broad, whereas the maxima for the sintered and un-sintered powders were 3442.3 cm^−1^ and 3445.2 cm^−1^, respectively, and they were attributed to the hydrogen-bonded water molecules and hydroxyls. According to a study conducted by Zakharova *et al.* [35], the thermally-treated powder had strong vibrations that were detected at 588.2, 854.3, 995.1, 1384.6, and 1644 cm^−1^, associated respectively with the stretching mode of oxygen in the Mo–O–Mo units and the Mo=O stretching mode, which specifies a layered orthorhombic α-MoO_3_ phase.

**Figure 3:**
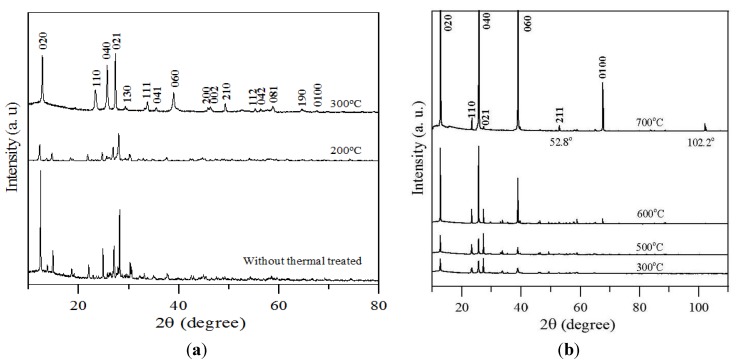
(**a**) XRD characteristics of molybdic trioxides without thermal treating and at sintering temperatures up to 300 °C; (**b**) XRD characteristics of molybdic trioxides at sintering temperatures from 300 to 700 °C.

There have been no studies concerning the mechanisms associated with the reaction of ammonium molybdate tetrahydrate with ethylene glycol to produce *α*-MoO_3_. However, no complete explanations of the potential reaction mechanisms were available. Nevertheless, the mechanisms according to results of related research can associate with all this reaction. Morris [36] reported that the oxidation of ethylene glycol to acetaldehyde and to acetic acid as shown in Equations (2) and (3). The acetic acid decomposes to form acetate ions (CH_3_COO^−^) and hydrogen ions (H^+^), as shown in Equation (4). The ammonium molybdate tetrahydrate solution was converted to the hydrated form, which consisted of hydronium ions (H_3_O^+^) and heptamolybdate (Mo_7_O_24_^−6^), as shown in Equation (5). Then, Mo_7_O_24_^−6^ and H^+^ (from Equation (4)) reacted to form molybdenum trioxide (MoO_3_·H_2_O), as shown in Equations (6) and (7) [37]. The structure of MoO_3_·H_2_O can be demonstrated by XRD dates as shown in Figure 3a. In this study, the molybdenum trioxide powder, when sintered at 300 °C and above, can form α–MoO_3_, as shown in Equation (8) that can demonstrated by XRD dates as shown in Figure 3a. The ammonium molybdate tetrahydrate has an ammonium group that formed NH_3_ gas at the high temperatures, and the mechanism is shown in Equation (9) [32].

2CH_2_OHCH_2_OH + O_2_ → 2CH_3_CHO + 2H_2_O (2)

2CH_3_CHO + O_2_ → CH_3_COOH + 2H_2_O (3)

CH_3_COOH → CH_3_COO^−^ + H^+^ (4)

(NH_4_)_6_Mo_7_O_24_·4H_2_O →Mo_7_O_24_^−6^ + 6NH_4_^+^ + 4H_2_O (5)

Mo_7_O_24_^−6^ + 3H^+^ → (H–O)_3_Mo_7_O_23_^ −3^ (6)

(H-O)_3_Mo_7_O_23_^−3^ + 3H^+^ → (H–O)_3_Mo_7_O_18_ → 7MoO_3_·H_2_O (7)

MoO_3_·H_2_O + O_2_ → α-MoO_3_ (8)

NH_4_^+^ → H^+^ + NH_3_ (9)

**Figure 4:**
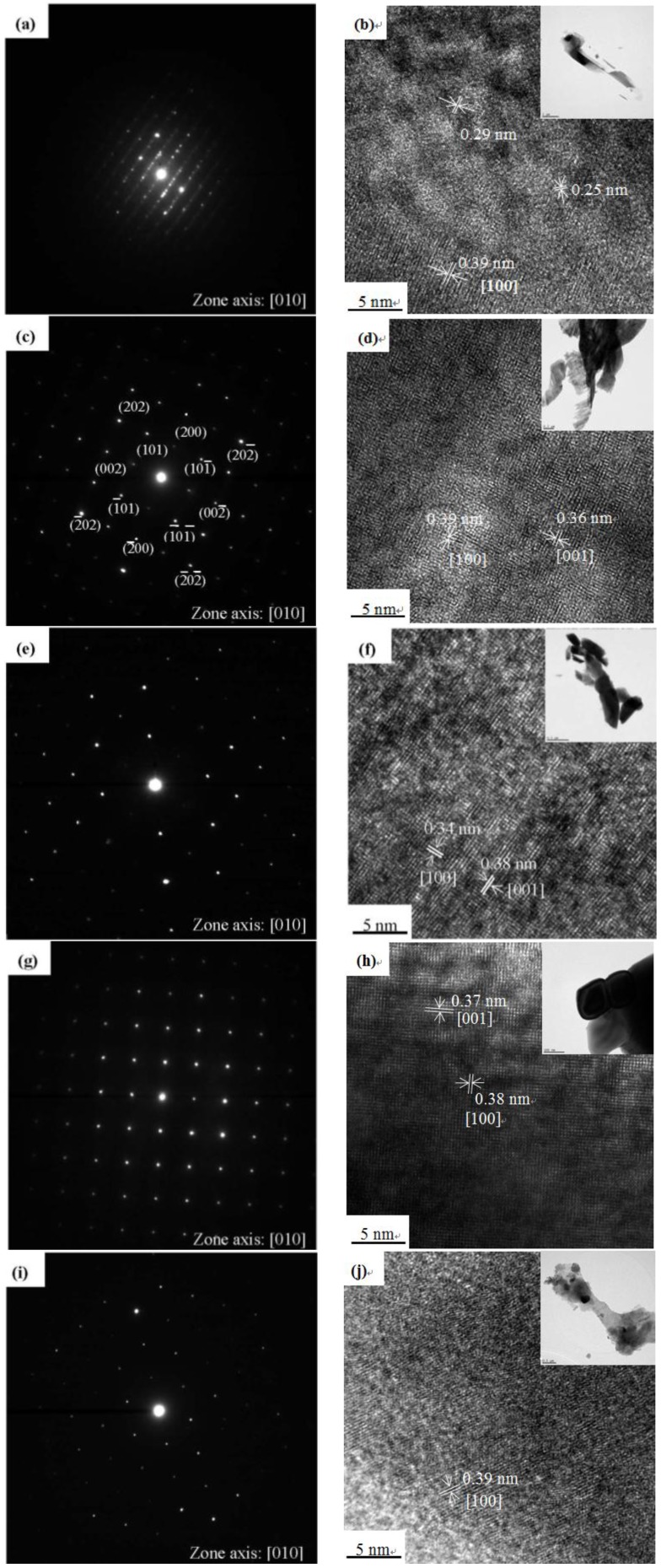
(**a**) SAED pattern and (**b**) HRTEM image of the molybdenum trioxides sintered for 1 h at 200 °C; (**c**) and (**d**) 300 °C; (**e**) and (**f**) 500 °C; (**g**) and (**h**) 600 °C; (**i**) and (**j**) 700 °C.

Figure 3a shows the XRD results before sintering and after sintering for 1 h at 300 °C. The figure shows that the powder that had not been sintered had XRD peaks, indicating that it had the same XRD characteristics as commercial molybdic acid powder (MoO_3_·H_2_O) [28], and, by referring to the JCPDS cards, it was difficult to determine the phases of the sample based on known literature data. Therefore, we conjectured that the white powder that was formed was MoO_3_·H_2_O that the powder was covered with poor crystalline and polycrystalline areas. When the powder had undergone sintering at 200 °C for 1 h, the positions of the XRD peaks were the same as those of the un-sintered powder, which indicated that the sintering at 200 °C for 1 h did not change the structure of the powder. But the TEM images and SAED patterns revealed that some areas of the powder had transitioned to an intermediate structure between polycrystalline and crystalline, as shown in Figure 4a,b. Therefore, we conjectured that a sintering temperature of 200 °C was approximately the temperature at which the structure of the MoO_3_ powder changes from the polycrystalline structure to the crystalline structure.

When the MoO_3_ powder was sintered for 1 h at temperatures ranging from 300 to 700 °C, its crystallinity and its peaks conformed to those of MoO_3_ for JCPDS database number 05-0508, as shown in Figure 3b. Figure 3b shows that the position and intensities of several main diffraction peaks for sintering temperatures from 300 to 700 °C are highly similar to those of the reference card (JCPD standard cards 05-0508) [38]. These peaks correspond to the orthorhombic phase of α-MoO_3,_ since the plane (020) peaks at 2θ of 12.74° clearly were detected, indicating the presence of the orthorhombic phase instead of the monoclinic phase [10]. It was indexed to correspond with the (002) and (200) crystallographic planes, which were specified as orthorhombic α-MoO_3_ [39]. The planes (110), (040), (021), (111), and (060) correspond to orthorhombic crystal symmetry and to the calculated lattice parameters (Å) of a, b, and c using the plane spacing equation for the orthorhombic phase for sintering temperatures between 300 and 700 °C. All the lattice parameters corresponded to space group Pbnm (no. 62) [40] of α-MoO_3_ for the various sintering temperatures. Furthermore, both lattice parameters, a and b, increased when the MoO_3_ powder was subjected to increasing sintering temperatures, which means that the powder’s growth was to the (101) direction. For the sintering temperatures from 300 to 700 °C, there was an increase in the intensity of the (0k0), such as (020), (040), (060), and (0100), and there was a substantial decrease in the intensity of the (021) face, indicating the effect of sintering at the various temperatures on crystal growth, as shown in Figure 3b. The results were same as those reported by Song *et al.* [41].

The crystallite sizes were estimated by using Equation (10), which is known as the Sherrer formula [42]:

(10) $$G_{hkl}=\frac{k\lambda }{\beta \cos(\theta )}$$

where *G_hkl_* is the average linear dimension of the crystal perpendicular to the diffracting plane (*hkl*), *β* (radians) is the full-width at half-maximum in the 2θ scan, *k* is a constant (0.89), *λ* is the wavelength of the X-rays (1.54 Å for Cu K_α_), *G_hkl_* is the diameter of the particles, and *θ* is the angle of the diffraction peak. Using this equation, it is possible to calculate the grain size by considering each sample’s major peak (020) acquired by XRD. The calculated crystallite sizes were 22.8, 38.8, 77.7, and 78.1 nm for the powders that were sintered for 1 h at 300, 500, 600, and 700 °C, respectively. It can be noted that the grain size of the sintered MoO_3_ powder increased was the sintering temperature increased from 300 to 700 °C. This implies that sintering temperatures over 300 °C produce more crystalline α-MoO_3_ powder and increase the grain sizes of the MoO_3_ crystallites.

At the sintering temperature of 700 °C, the α-MoO_3_ phase had an orthorhombic structure and higher crystallinity. Also at this temperature, the peaks with the largest intensities, *i.e.* (020), (040), (060), and (0100), occurred. These observations proved the existence of the lamellar structure, indicating the preferential growth of the oxide in the (0 2*k* 0) directions, confirming what the SEM images of lamellar structure shown in Figure 1 had indicated before. The most important structural characteristic of α-MoO_3_ is its structural anisotropy, which can be considered as a layered structure parallel to (010). These layers were stacked alternately along the (010) direction where the van der Waals interaction is the major binding force between the MoO_6_ octahedra layers, thereby forming α-MoO_3_, these results were same as those reported by Pereira *et al.* [29]. Each layer consisted of two sub-layers of distorted MoO_6_ octahedra to give three crystallographically-equivalent oxygen sites [43]. One might take advantage of the intrinsic structural anisotropy of α-MoO_3_ for tuning its properties by using sintering to grow α-MoO_3_ belts that have a strong preferred orientation. The newly formed peaks were located at 2θ = 52.8° and 102.2°, respectively.

The structural characteristics also were demonstrated by TEM. Figure 4c–J show the typical TEM observation of the MoO_3_ powder and the SAED pattern recorded perpendicular to the growth axis of the powder was composed of a highly-ordered diffraction lattice and a homogeneous array of diffraction dots for sintering temperatures from 500 to 700 °C for 1 h in air. In Figure 4c, the diffraction lattice planes of the SAED patterns indicate that the α-MoO_3_ powder had a single-crystal structure after undergoing thermal treatment at 300 °C for 1 h. It can be indexed to an orthorhombic α-MoO_3_ phase with a zone axis along the (010) direction. This implies that preferential growth occurred along the *c*-axis or (001) direction, implying that the MoO_3_ powders were well-crystallized and single crystallites. These observations are consistent with the XRD results (Figure 3) that demonstrated a high degree of crystallinity. In addition, the clear lattice stripes of the HRTEM image in Figure 4d show that the crystal had well-defined (100) and (001) planes of α-MoO_3_ single-crystal structure with the lengths of the lattice stripes being about 0.39 and 0.36 nm, respectively. These results were the same as those reported in Li *et al.* [44]. The SAED and HRTEM images of sintering temperatures from 500 to 700 °C are shown in Figure 4e–j. The diffraction pattern arrangement was the same as that in the SAED images, but the distances of the HRTEM images were a little different, and they were not coordinated with the lattice stripes of α-MoO_3_, *i.e.*, 0.39 and 0.36 nm, which could have been caused by some defects in the structure. 

The chemical compositions of the un-sintered powder and the powders sintered at various temperatures were investigated by XPS spectroscopy. The molybdenum oxidation state was estimated by deconvolution of the peaks in the Mo 3d region. The Mo 3d spectrum typically consisted of a Mo 3d_3/2_–Mo 3d_5/2_ doublet due to the spin–orbit coupling. The components were spaced by a difference of 3.13 eV. The doublet was Gaussian–Lorentzian shaped. The deconvolution was performed using Gaussian-Lorentzian sum function with 20% Gaussian and 80% Lorentzian values [45]. According to a study by Deng *et al.* [46], the Mo 3d region was fitted by doublets with fixed spectroscopic parameters, such as doublet of a hexavalent molybedum at 232.76 and 235.88 eV, which correspond to the Mo 3d_3_*_/_*_2_ and 3d_5_*_/_*_2_ orbitals, respectively, spin-orbit separation (3.1 eV), Mo 3d_3/2_ to Mo 3d_5/2_, intensity ratio (0.66), and full width at half maximum (FWHM) of 1.7 eV, but with independent and variable positions and intensities as optimized by the program.

**Figure 5:**
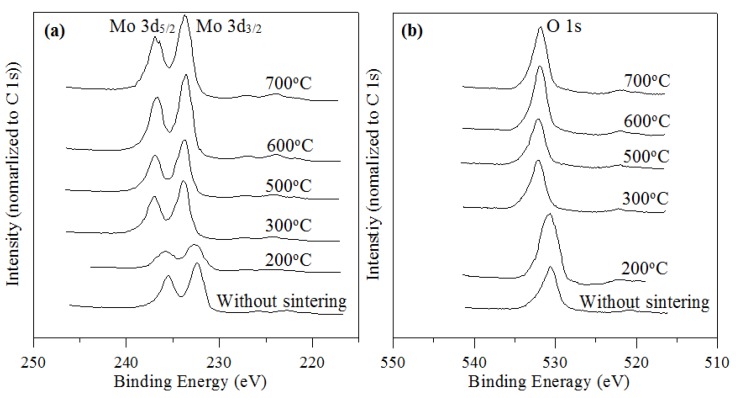
XPS characteristics of (**a**) Mo and (**b**) O of molybdenum trioxides for various sintering temperatures.

Figure 5a shows the peaks with binding energies of Mo 3d_5/2_ and Mo 3d_3/2_ of the MoO_3_ powders before sintering and after sintering at various temperatures. The binding energies of Mo 3d_5/2_ and Mo 3d_3/2_ for the un-sintered powder were located at 232.49 eV and 235.46 eV, respectively. At a sintering temperature of 200 °C, at which the binding energies of Mo 3d_5/2_ and Mo 3d_3/2_ were 232.69 and 235.87 eV, respectively, there was a minor decrease in the standard value, and the energy peaks were lower than samples that were sintered at 300 °C and above, as shown in Table 2. The lower energy peaks were associated with the lower oxidation state of the Mo^6+^ ions, which tends to result in lower binding energies, as stated previously [47]. For the two peaks of the powder that was sintered at 300 °C for 1 h, the binding energies of the Mo 3d_5/2_ peak and the 3d_3/2_ peak were shifted to 233.65 eV and 236.92 eV, respectively, which was attributed to the higher oxidation state of Mo^6+^ [41]. For sintering temperatures from 500 to 700 °C, the binding energies of the Mo 3d_5/2_ and the 3d_3/2_ peaks were almost same as those for a sintering temperature of 300 °C. However, when the sintering temperature was 300 °C and above, the binding energies were about 1 eV greater than the binding energies without sintering. In Table 2, the spin-orbit splitting of all various sintering temperatures used for the MoO_3_ powders between Mo 3d_5/2_ and Mo 3d_3/2_ signals were set to 3.03–3.32 eV, implying that the single-crystal surface probably was terminated with an O layer [39]. The FWHM and intensity ratio values of all MoO_3_ powders that were sintered at various temperatures also are given in Table 2.

**Table 2:** XPS results for different sintering temperatures.

Sintering Temperature (°C)	Mo 3d_5/2_ (eV)	FWHM (eV)	Mo 3d_3/2_ (eV)	FWHM (eV)	ΔMo 3d (eV)	O 1s (eV)	FWHM (eV)	O/Mo atomic ratio	intensity ratio
Before sintering	232.46	1.57	235.49	1.42	3.03	530.59	2.19	4.1	0.62
200	232.69	1.95	235.87	1.9	3.27	530.67	2.57	4.9	0.62
300	233.65	1.41	236.92	1.41	3.27	532.08	2.04	3.6	0.61
500	233.72	1.45	236.93	1.39	3.21	532.08	1.94	3.4	0.68
600	233.55	1.5	236.77	1.5	3.22	531.85	1.94	3.3	0.57
700	233.61	1.58	236.93	1.64	3.32	531.81	2.08	3.1	0.6

The XPS results also demonstrated that the structures were α-MoO_3_, which possesses oxygen vacancies. The O 1s peak in the XPS spectrum of the powder structures is shown in Figure 5b. The peaks of O 1s of the oxygen atoms that were bound to Mo were at 530.59 eV and 530.67 eV for the powder that had not been sintered and the powder that had been sintered for 1 h at 200 °C, respectively. The values being located in the range of 530.5 to 531.1 eV that mean the ionization characteristics of the oxygen species were integrated in the material as OH^−^ or O^2−^ [48], which was attributed to the presence of crystal bulk oxygens [47]. This resulted in the shifting of the peak from 532.08 to 531.81 eV for O^2−^ of the powders that had been sintered at temperatures from 300 to 700 °C, indicating an increase in the overall crystallinity of the particles of powder [49] and the presence of O^2−^ in the oxygen-deficient regions within the matrix of the MoO_3_ [50]. They can be associated with sites at which the coordination number of oxygen ions is smaller than in a regular site that has a higher covalence of the M–O bonds [48].

**Figure 6:**
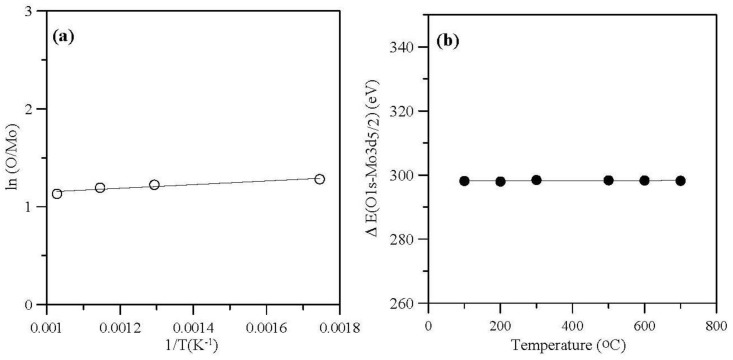
(**a**)Plot of ln(O/Mo) *versus* 1/T for sintering temperatures of molybdenum trioxides powder from 300 to 700 °C; (**b**) Δ(O 1s–Mo 3d_5/2_) values for various sintering temperatures.

Table 2 shows the dependence of the surface concentration ratio, O/Mo, on sintering temperature. The O/Mo atomic ratio decreased, which results indicate that the MoO_3_ was partially reduced to an oxidation state between Mo^5+^ and Mo^6+^ as the sintering temperature increased (200 to 700°C). In addition, Figure 6a shows the plot of ln(O/Mo) *versus* 1/T for sintering temperatures of MoO_3_ powder from 300 to 700 °C. 

The results present an Arrhenius-type behavior, with an activation energy of 0.016 eV for the process. The presence of high density grain boundaries that have high-diffusivity pathways is the reason for the low activation energy. Figure 6b shows Δ(O 1s–Mo 3d_5/2_) values for various sintering temperatures, which average of the values is about 298.2 eV. The value was lower than for the clean Mo metal, 303.2 eV [51,52]. These results are related to the chemical shift of Mo toward higher binding energies during the oxidation process. 

### 2.3. Effect of Sintering Times

**Figure 7:**
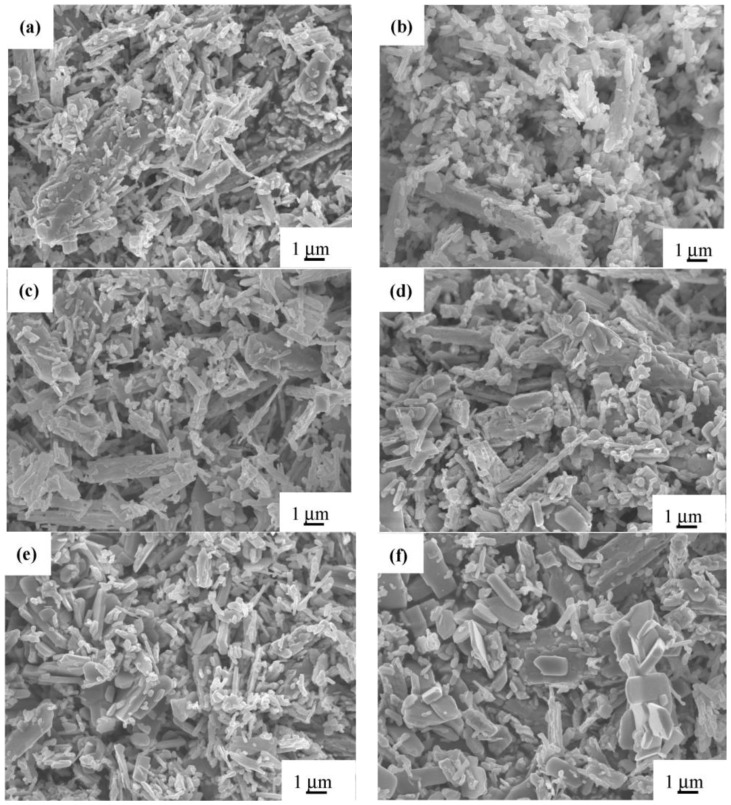
SEM images of molybdenum trioxide powders sintered a temperature of 500 °C for sintering times of (**a**) 0.5 h; (**b**) 1 h; (**c**) 2 h; (**d**) 3 h; (**e**) 4 h; (**f**) 5 h.

**Figure 8:**
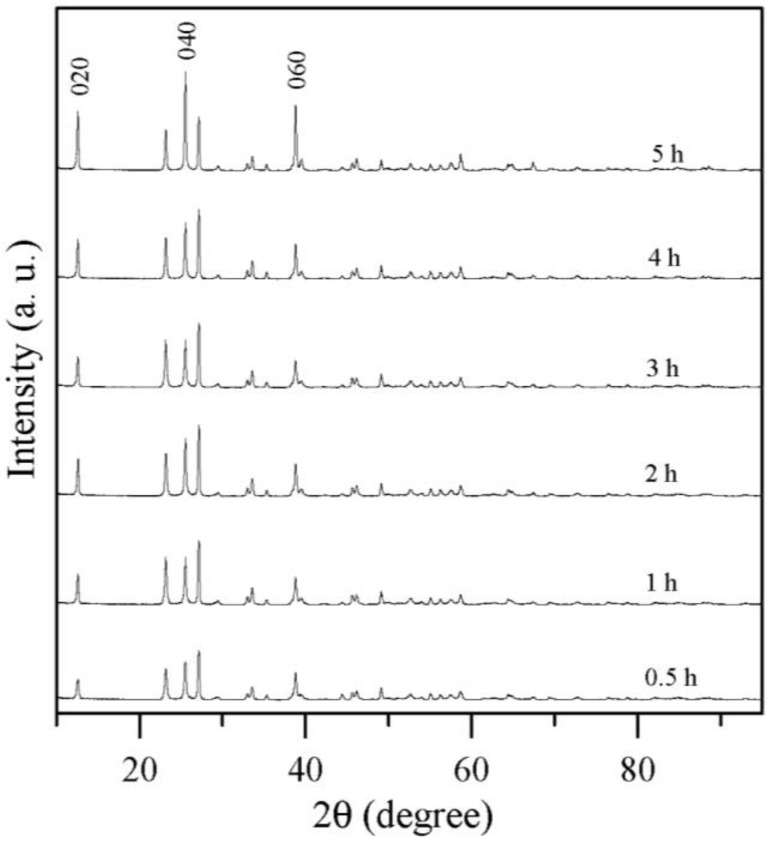
XRD characteristics of powders sintered at 500 °C for various sintering times.

Sintering times have an effect on the formation of α-MoO_3_ powders. At a sintering temperature of 500 °C, the powder had the smallest particle size of powder than at any other sintering temperature that was tested, and the SEM images are shown in Figure 1d. Therefore, the results of our study of α-MoO_3_ powders that underwent sintering at 500 °C for 0.5, 1, 2, 3, 4, and 5 h are shown in the SEM images in Figure 7. The results indicated that the particle size was larger for a sintering time of 0.5 h than it was for 1.0 h. However, as the sintering time was increased from 2 to 5 h, the particle sizes of the α-MoO_3_ increased. At a sintering time of 5 h, the shapes of the particles of powder were rectangular because of the increase in intensity of the (020), (040), and (060) in XRD, as shown in Figure 8. Therefore, a sintering time of 1 h at 500 °C produced the smallest particles, *i.e.* in the range of 200–300 nm.

## 3. Experimental Section

The ammonium molybdate tetrahydrate ((NH_4_)_6_Mo_7_O_24_·4H_2_O) was supplied by Sigma-Aldrich Co. Ltd. (St. Louis, MO, USA). The reacting agent was ethylene glycol with a purity of 99.5%, which was provided by Showa Chemical Co. Ltd.

Fifty milliliters of ethylene glycol were mixed with 250 mL of 0.1 M ammonium molybdate tetrahydrate solution. The mixture was heated for 40 min at 120 °C, producing a precipitate and a dark blue solution. After cooling, the products were centrifuged three times for 5 min each time at a speed of 5000 rpm. After centrifugation, the dark blue solution was poured off, and the precipitate was washed with distilled water, placed in a dish, and placed in the oven at 80 °C to 24 h. The result was white molybdenum trioxide powder. The white powder was treated at different sintering temperatures for 1 h.

Analyses of the surface morphologies of the samples were conducted by scanning electron microscopy (SEM) with electron dispersion spectroscopy characterization using a JEOL JED 2300 instrument. X-Ray diffraction (XRD) patterns of the solid samples were recorded in a Rigaku (Japan) TTRAX III rotating anode diffractometer with a Ni-filtered, Cu-K radiation source (wavelength of 1.54 Å). The patterns were identified as α-MoO_3_ by comparing them to JCPD standard cards (05-0508). Fourier transform infrared (FTIR) spectra were obtained with a JASCO FT/IR-470 plus spectrometer in the wavelength range from 400 to 4000 cm^−1^ and with a resolution of 4 cm^−1^ for each spectrum. The selected area electron diffraction (SAED) of the samples was analyzed by transmission electron microscopy (TEM) on a JEOL 2100 F transmission electron microscope. High-resolution transmission electron microscopy (HRTEM) images were obtained using a JEOL 2100 F microscope at an accelerating voltage of 200 kV. The TEM specimens were prepared by suspending the samples in distilled water and placing small droplets of those solutions onto a standard, carbon-supported, 600-mesh, copper grid, which was placed into oven at 100 °C for 1 h to dry the samples. 

X-ray photoelectron spectroscopy (XPS) was used to determine the chemical bonding state and surface composition of the samples. The XPS spectrometer (Microlab 350) consisted of an X-ray source, which consisted of Al Ka radiation (1486.6 eV) in ultra-high vacuum (2.00 × 10^−9^ torr) at room temperature and an area larger than 5 mm^2^. The energy resolution of the instrument was 0.16 eV. The C 1s peak (284.8 eV) was used as the internal standard for binding-energy calibration. 

## 4. Conclusions 

We demonstrated that ethylene glycol can be used to react with ammonium molybdate tetrahydrate, producing α-MoO_3_ after the precipitate produced in the reaction is sintered at a temperature of 300 °C for 1 h. This process was easier and simpler than other processes that have been proposed. The particle size was about 200–300 nm, and the particles of powder were rice-shaped when the sintering temperature was 500 °C. At a sintering temperature of 700 °C, the powders were in the form of piled sheets of nanometer-scale thicknesses. When the powder was sintered at temperatures ranging from 300 to 700 °C, there was an increase in intensity of the (0k0), such as (020), (040), (060), and (0100), and there also was a substantial decrease in intensity of the (021) face. The grain size of sintered MoO_3_ powder increased as the sintering temperature increased from 300 to 700 °C. The lattice stripes of the HRTEM image demonstrated that the crystal had well-defined (100) and (001) planes of α-MoO_3_ single-crystal structure, with the lengths of the lattice stripes being about 0.39 and 0.36 nm, respectively. XPS results also demonstrated that the structures were α-MoO_3_. For sintering temperatures of the MoO_3_ powder ranging from 300 to 700 °C, the low activation energy of only 0.016 eV was observed, resulting from high-density grain boundaries that had high-diffusivity pathways. Of the sintering times that were tested, the time of 1 h when the sintering temperature was 500 °C produced the smallest particle size of the α-MoO_3_ powder. 
